# Biomonitoring of Mercury in Hair among a Group of Eritreans (Africa)

**DOI:** 10.3390/ijerph17061911

**Published:** 2020-03-15

**Authors:** Maria Luisa Astolfi, Carmela Protano, Elisabetta Marconi, Lorenzo Massimi, Daniel Piamonti, Marco Brunori, Matteo Vitali, Silvia Canepari

**Affiliations:** 1 Department of Chemistry, Sapienza University, Piazzale Aldo Moro 5, I-00185 Rome, Italy; l.massimi@uniroma1.it (L.M.); silvia.canepari@uniroma1.it (S.C.); 2 Department of Public Health and Infectious Diseases, Sapienza University, Piazzale Aldo Moro 5, I-00185 Rome, Italy; carmela.protano@uniroma1.it (C.P.); elisabetta.marconi@uniroma1.it (E.M.); matteo.vitali@uniroma1.it (M.V.); 3 Department of Cardiovascular, Respiratory, Nephrology, Anaesthesiology and Geriatric Science, Sapienza University, Viale del Policlinico 155, I-00161 Rome, Italy; daniel.piamonti@gmail.com (D.P.); marco.brunori@uniroma1.it (M.B.)

**Keywords:** biological monitoring, hair analysis, toxic element, human health

## Abstract

Small-scale or artisanal mining, using gold-mercury amalgamation to extract gold from ore, is a significant source of exposure for the workers and nearby populations. Few studies on hair mercury (Hg) have been conducted in Africa despite the fact that Africa has several gold deposits. No studies have been conducted in Eritrea that is one of the emerging gold producing countries in Africa. The aim of the study was to assess the Hg concentration in hair samples (*n* = 120) of a population living in Asmara, capital of Eritrea, and to evaluate the influence of some factors on the Hg levels in hair. Information on age, height, weight, occupation, smoking and fish consumption of participants were collected via questionnaire. Hair Hg concentration was significantly higher among women compared to men (*p* < 0.001) and among women preparing spicy products in Medeber market compared to those who did other jobs (*p* = 0.010). These results highlight the need for routine biomonitoring surveys and for health promotion campaigns devoted to local decision makers and workers.

## 1. Introduction

Mercury (Hg) is a naturally occurring element that can be distributed in the environment by both natural processes and human activities [1]. Hg is recognized as a dangerous environmental pollutant due to its high persistence in the environment and its toxicity to several biological species, including humans [2,3,4]. The entire global population is potentially exposed to Hg due to its long-range transport and its ubiquity in global marine ecosystems [5]. Hg can be found in three main forms: elemental, inorganic and organic (such as methylmercury, MeHg), each with their own implications for human health [6,7]. People may be exposed to elemental or inorganic Hg through inhalation of ambient air, during occupational activities and from dental amalgams [8], but the main nonoccupational exposure of humans to Hg is in its organic form (MeHg), considered the most toxic species of Hg [9]. The main route of exposure to MeHg is through diet, by the consumption of fish and seafood [10,11]. Small-scale or artisanal mining, using gold-mercury amalgamation to extract gold from ore, is a significant source of exposure for the workers and nearby populations [12,13]. Besides, metallic Hg wastes are usually dumped into or near watercourses. These discharges can lead to high MeHg levels in fish of these water bodies [14]. In this way, Díez et al., [15] found that mean Hg levels were greater in people living close to industrial mining activity sites compared with a site placed hundreds of kilometers away. To our knowledge, in Africa, the impact of Hg contamination has not been thoroughly studied and the human exposure assessment has been very limited, despite the fact that artisanal and small-scale gold mining continues to expand and to increase in some hotspots in African countries, in response to the global demand for gold [12,16,17,18].

Exposure to toxic Hg levels can cause neurological, renal, cardiac and reproductive disorders as well as genetic damage [19,20,21]. Human exposure to Hg can be assessed using an environmental monitoring approach. In this case, the Hg level in specific matrices such as food, water and air can be determined and external exposure to Hg estimated [11]. However, this method requires a lot of data on Hg levels in different matrices and does not allow one to have information on the real dose absorbed by the human body (body burden) [22]. For this reason, the biological monitoring approach is currently recognized as a more appropriate tool for assessing the exposure and the related risks for human and environmental health [23,24,25,26,27,28]. Hg concentrations in blood and hair have been used as biomarkers to assess the human exposition to this element [29]. The total Hg in human hair is a good indicator of long-term Hg exposure, particularly for methylmercury [30]. The hair-to-whole blood ratio has been estimated to be about 250 to 1 [30,31]. In this way, hair samples appear as an interesting alternative because it is a noninvasive matrix and it is easy to sample [32,33,34].

In the present study, we assessed the exposure to Hg in the hair of a group of residents in Asmara, capital of Eritrea (Africa), due to the possible contamination by small-scale or artisanal extraction of gold from deposits present in a few kilometers away from Asmara, and due to the processing of metal objects (for men) and spices (for women) at the Medeber market. We also compared the results with similar international studies and performed an analysis of the main exposure predictors. The risk was characterized by comparing the Hg levels in hair using international health-based guidance values. 

## 2. Materials and Methods

### 2.1. Study Design and Sample Collection

This study included 120 adult hair samples selected in Asmara, the capital of the small East African country of Eritrea. The subjects were randomly selected from a larger population of 840 subjects participating in 2017 in the “Medeber project”. This project, carried out in collaboration with the Italian nonprofit organization “ASS.ITER. Onlus”, includes intervention and epidemiological studies focused on Eritrean adults. In particular, about 53% of the volunteers (*N* = 64 individuals; 32 males and 32 females) were employed at the Medeber market in Asmara (Table 1). In this market, men hammer, cut, weld and transform metal objects and old machinery into something useful, while women prepare “berbere”, a mixture of spices (like peppers, ginger, cardamom, coriander and cloves), and other spicy products [35].

The study was approved by the Minister of Health of Eritrea through acknowledgement of the ASS.ITER. Onlus, within the “Medeber project” and it was carried out according to the Declaration of Helsinki. All the recruited individuals received clear information on the objectives and the phases of the project, including the anonym treatment of the collected human scalp hair samples and information. In addition, the methods used to protect the identity and privacy of each participant were clearly explicated. A cultural linguistic mediator was present during all the phases of the project involving Eritrean individuals in order to guarantee the complete understanding of all information on research project. The individuals who decided to participate donated the hair sample and filled out an anonym questionnaire. No personally identifying information was collected for assuring the anonymity of each participant.

After washing the hair of each volunteer with shampoo and drying using a previously reported method [36,37], hair samples (0.1 g; 2 cm) were collected by cutting hair from the occipital region of the head in the same place for both males and females. Stainless steel scissors and disposable vinyl gloves were used to avoid contamination. The hair samples were then placed into polyethylene bags, transported to the laboratory, and stored in the dark at room temperature (21 °C) until further analysis.

### 2.2. Chemical Analysis

Hg exposure for 120 individuals was estimated by measuring total Hg content in hair samples. These were trimmed in order to analyze the 2 cm length of hair closest to the scalp. Hair grows at an average rate of one cm per month [38] and, thus, our analysis was intended to estimate the total Hg exposure for the two-month period preceding analysis. Hair total Hg content was determined by thermal decomposition-gold amalgamation atomic absorption spectroscopy, as described in a previous paper [39]. Briefly, when the sample arrived to the laboratory, the hair strands were further cut into smaller pieces (3–5 mm) using stainless scissor. Then, 5 mg of the sample were placed in a nickel boat for Hg determination in the Advanced Mercury Analyzer (AMA-254, Altec Ltd., Prague, Czech Republic). Each sample was analyzed in duplicate and average mean concentrations of total Hg (µg g^−1^) was used for data treatment in the statistical analysis. Standard stock solution of Hg (1002 ± 7 mg L^−1^; SCP Science, Baie D’Urfé, Canada) and deionized water (resistivity ≤18.3 MΩ cm) generated by an Arioso Power I RO-UP Scholar UV deionizer (Human Corporation, Songpa-Ku, Seoul, Korea) with 1% HNO_3_ v/v (assay >67%; residue <1 mg L^−1^; Promochem, LGC Standards GmbH, Wesel, Germany) were used to prepare calibration standards. The instrument calibration was verified by the analysis of a hair standard reference material (ERM-DB001; sample no. 0196; Joint Research Centre, Geel, Belgium) every 10 samples in a batch run. Accepted measurements were within 10% of certified value. The detection limit was 0.07 ng Hg.

### 2.3. Data Analysis

Information about the participants was collected by a questionnaire specifically designated for the present study. Questions were translated into Tigrinya (the language spoken in Asmara) and validated before its administration to the studied individuals. Note that, due to the often-illegal nature of mining in the area, we chose not to ask participants about their mining activities. In particular, we collected the following characteristics of each participant: gender, age, height and weight, smoking habits and fish consumption. Then, we coded the collected information and entered it into a database created “ad hoc”. Gender was categorized as 0 = male and 1 = female; age was entered as the number of years and used as a continuous variable; smoking habits were coded as 0 = never smoker, 1 = current smoker and 2 = ex-smoker; and fish consumption was coded as 0 = Never/Seasonally/Sometimes, 1 = weekly and 2 = daily. We also created the categorical variable “age groups” as follows: adolescence (15–21 years old), early adulthood (22–39 years old), second adult age (40–59 years old) and third age (≥60 years old). Body mass index (BMI) was calculated from participants’ weight and height. BMI was then used both as a continuous variable and as a recoded categorical variable as follows: underweight = 0, normal weight = 1 and overweight/obesity = 2. The occupational setting of each participant were coded as “not working at Medeber” = 0 and “work at Medeber” = 1.

Statistical analyses were performed by the use of IBM SPSS Statistics 25 software (IBM Corp., Armonk, NY, USA). First of all, we elaborated descriptive statistics, reporting categorical variables as absolute and relative frequencies while continuous variables as arithmetic mean (AM) ± standard deviation (SD) and range (minimum and maximum) value. For evaluating the influences of the collected variables on hair Hg concentration, we preliminary assessed the normality Hg level by use of the Kolmogorov–Smirnov test. The results demonstrated that natural log-transformed data were normally distributed. Thus, t-tests for independent samples were used to test the statistical significance of the differences in the mean levels of Hg according to gender and occupational scenario. Besides, a one-way ANOVA test was used to assess differences in the Hg level based on age groups or BMI categories as categorical variables. Finally, Pearson’s correlation coefficients were used to evaluate the association between the Hg level and the age or BMI (continuous variables) of each participant. The significance level for all tests was p ≤0.05 (two-tailed).

## 3. Results

The characteristics of the study population are shown in Table 1. A total of 120 participants were recruited, with a similar proportion of males (45.8%) and females (54.2%). The largest age group was those 22–39 years old (39.0%). Regarding the occupational scenario, just over half of the individuals worked at the Medeber market (53.3%), while just under half of the individuals did not work at Medeber. BMI mean value (21.2 ± 4.0) corresponded to a normal weight (18.5–24.9). A majority of the population has never smoked (90.8%) and consumed fish never, or just seasonally or sometimes (94.2%). Therefore, we decided not to consider these variables in the subsequent statistical elaboration. 

The results of the univariate statistical analyses according to gender, occupational scenario, age and BMI groups are shown in Table 2 and Table 3, while the results of the association between the Hg level and the age or BMI (continuous variables) of each participant are reported in Table 4. Significant differences were found in the mean levels of Hg according to gender and occupational scenario. Indeed, the Hg levels were significantly higher in women (2.1 ± 4.3 µg g^−1^) than in men (0.23 ± 0.37 µg g^−1^; *p* < 0.001; Table 2) and in women who worked in the Medeber market (3.6 ± 6.0 µg g^−1^) compared to those obtained in all others (*p* < 0.010; Table 3). No significant correlations between hair levels of Hg and BMI or age were found.

## 4. Discussion

In the last years, several studies conducted in European countries [40,41,42,43,44,45], USA [46], Canada [47], Japan [48] and China [49] demonstrated that exposure to Hg is still a crucial public health concern, which no one country can solve alone. In 2013, the Minamata Convention adopted comprehensive measures to eliminate or at least reduce the sources of emission and exposure to Hg (http://www.mercuryconvention.org). Currently, the collaboration between the World Health Organization (WHO) European Center for Environment and Health and the United Nations Environment Program (UNEP) aims to plan human biomonitoring at a global level as a key tool to evaluate the baseline conditions of human exposure to Hg and to reduce this exposure and the relevant risks [8].

In this study, hair Hg concentrations were below the WHO No Observed Adverse Effect Level (NOAEL; 50 μg g^−1^) [30], however in 26.7% of people exceeded the United States Environmental Protection Agency (USEPA) reference dose of 1.2 μg g^−1^ [38,50]. In addition, the Hg levels in some hair samples of women also exceeded the NOAEL (10 μg g^−1^) associated with fetus neurotoxicity [51,52]. Communities near mining may have unexpected dietary sources or can be exposed to other Hg species [12,53]. Since the Hg in hair is typically dominated by MeHg species [32], diet is often presumed to be the dominant exposure route; however, a fraction of Hg in hair could be the inorganic form caused by other dietary and non-dietary routes (e.g., inorganic Hg exposure from gold amalgamation) [12,54]. Laffont et al. [54] found that modeled inorganic Hg levels in hair samples correspond well to measured inorganic Hg concentrations, demonstrating that inorganic Hg exposure sources to gold miners can be monitored by the use of hair samples. Although there are more appropriate biomarkers to assess inorganic Hg [55], we chose to use hair because it is a stable matrix with easy collection, transport and storage [33]. This is very important when considering biomonitoring in developing countries and remote areas where immediate sample cooling to 4 °C or even freezing below −18 °C to prevent degradation and to reduce bacterial growth is not available [33,34]. Although the urine matrix is the standard biomarker to assess the exposure to inorganic Hg through a non-invasive approach, urinary sediments tends to precipitate during the storage period. Trace elements in urine may coprecipitate, or adsorb onto the surface of the precipitates [56]. In particular, Hg was found to be concentrated in the precipitate of acidified urine after two days’ storage [56].

Our data indicate that some women who prepare spices in the Medeber market are exposed to higher Hg levels than others workers. Despite that the study population did not consume fish regularly, hair Hg concentration was significantly higher among women compared to men (*p* < 0.001) and among women preparing spicy products in the Medeber market compared to those who did other jobs (*p* = 0.010). Hair Hg concentrations in 53.1% of women employed in the Medeber market exceeded the USEPA reference dose of 1.2 μg g^−1^. In fact, plants can also contain Hg due to the bioaccumulation of Hg from soil, water and atmosphere [57]. In recent years, there has been a growing interest in monitoring heavy metal contamination of spices [57,58,59]. These can contain toxic elements in a wide range of concentrations, and their content can vary according to the location and the type of soil for cultivation, fertilizers, herbicides and water resources used for irrigation, climate and environmental pollution levels [58]. The levels of toxic elements (like As, Cd, Hg and Pb) in pepper, ginger and black cumin, reported in previous studies [58], exceed in many cases the maximum limits allowed regulated by different legislations [60,61] and recommendations by WHO [62,63].

Previous studies showed that gender is unlikely to be an important factor determining Hg accumulation in hair [64,65,66,67,68,69]. However, other studies found that males had higher mean values of Hg concentrations in hair than females in Italy and Spain [15,44]. These gender-related differences could be due to a higher fish consumption per serving in males than in females or the possible Hg elimination in females as a result of different hair treatments, such as artificial hair waving and hair coloring/dyeing [15].

The relationship of hair Hg concentration with age is of interest. Few studies have looked at the effect of age on hair Hg concentration [67,70,71,72,73,74,75]. Our results show that there were no significant correlations between Hg concentrations and age (*p* = 0.974, Table 4) in accord with other studies [67,71,72]. In contrast, other authors showed that the Hg level in hair increases with age [73,74,75].

Our finding that total hair Hg levels were statistically significantly higher in women preparing spices, supports the idea that anthropogenic activities increase the risk of Hg exposure and suggests that further studies include a consideration of nutritional, occupational and residential exposures.

Our study had several important limitations. First, the present study was a cross-sectional study, therefore, it did not allow an evaluation over time. Second, we did not know whether people engaged in mining took part in it. Third, we did not analyze the spices processed in the Medeber market. Fourth, we had no data on Hg levels in blood that would be useful for comparison with the data obtained in hair. Nevertheless, to our knowledge, it is important to note that this is the first human biomonitoring study performed on Eritrean individuals using hair samples to assess Hg exposure.

## 5. Conclusions

The results of the present study evidenced that gender was an important interfering factor on the hair concentrations of Hg. Consequently, the use of gender specific reference ranges was strongly recommended in human biomonitoring studies performing to assess human health risks related to Hg exposure. Furthermore, our findings indicate the relevance of the occupational scenario on the Hg exposure of the monitored population. This indicates the presence of high Hg sources in this site. As a result, health promotion campaigns are needed to increase health and safety protection strategies for the Medeber workers, such as the use of gloves and masks or other appropriated personal protective equipment. In addition, human biomonitoring studies would be needed to verify whether imposed controls, and health and safety promotions are effectives. These in-field data on Hg levels in hair matrix can be used as baseline information for future programs aimed to control environmental pollution, particularly in developing countries.

## Figures and Tables

**Table 1:** Characteristics of the studied population.

Variable	Studied Population Characteristics		Descriptives in % if not Stated Otherwise (*N*)
Gender	Male		45.8 (*N* = 55)
	Female		54.2 (*N* = 65)
	Unknown		0 (*N* = 0)
Occupation	Work at Medeber		53.3 (*N* = 32 males; *N* = 32 females)
	No work at Medeber:	Employed	19.2 (*N* = 9 males; *N* = 14 females)
		Unknown job	23.3 (*N* = 12 males; *N* = 16 females)
	Unknown		4.2 (*N* = 2 males; *N* = 3 females)
Smoking habit	Never smoker		90.8 (*N* = 109)
	Current smoker		4.2 (*N* = 5)
	Ex-smoker		0.8 (*N* = 1)
	Unknown		4.2 (*N* = 5)
Fish consumption	Never/Seasonally/Sometimes		94.2 (*N* = 113)
	Weekly		4.2 (*N* = 5)
	Daily		0 (*N* = 0)
	Unknown		1.6 (*N* = 2)
Body mass index (BMI) categories	Underweight (<18.5)		26.7 (*N* = 32)
	Normal weight (18.5–24.9)		57.5 (*N* = 69)
	Overweight/obesity (25–29.9/ 30 or greater)		11.7 (*N* = 14)
	Unknown		4.2 (*N* = 5)
Age groups	Adolescence (15–21 y)		10.0 (*N* = 12)
	Early adulthood (22–39 y)		65.0 (*N* = 78)
	Second adult age (40–59 y)		17.5 (*N* = 21)
	Third age (≥60 y)		4.2 (*N* = 5)
	Unknown		3.3 (*N* = 4)
Age			AM ± SD ^1^ = 33.6 ± 11.7 Min–Max = 15–76
BMI			AM ± SD ^1^ = 21.2 ± 4.0 Min–Max = 15.4–44.6

^1^ Arithmetic mean (AM) ± standard deviation (SD).

**Table 2:** Demographics of participants and hair mercury concentrations (μg g^−1^).

Variable	Studied Population Characteristics	AM ± SD ^1^	Median (Min–Max)	*p*-Value ^2^
Gender	Male	0.23 ± 0.37	0.10 (0.03–2.24)	< 0.001
	Female	2.1 ± 4.3	0.30 (0.01–21.4)	
	Overall	1.3 ± 3.3	0.16 (0.01–21.4)	
Work at Medeber	No	0.7 ± 1.8	0.13 (0.01–10.1)	0.132
	Yes	1.7 ± 4.3	0.16 (0.02–21.4)	
Age groups	Adolescence (15–21 y)	0.29 ± 0.36	0.13 (0.03–1.22)	0.680
	Early adulthood (22–39 y)	1.5 ± 3.9	0.16 (0.02–21.4)	
	Second adult age (40–59 y)	0.8 ± 2.0	0.13 (0.01–8.2)	
	Third age (≥60 y)	0.7 ± 1.0	0.27 (0.1–1.5)	
Body mass index (BMI) categories	Underweight (<18.5)	0.7 ± 1.7	0.09 (0.03–8.2)	0.774
	Normal weight (18.5–24.9)	1.4 ± 3.3	0.17 (0.01–21.4)	
	Overweight/obesity (25–29.9/ 30 or greater)	1.7 ± 5.6	0.14 (0.04–21.2)	

^1^ Arithmetic mean (AM) ± standard deviation (SD). ^2^ Male and female or work at Medeber and not work at Medeber were compared using unpaired *t*-test (natural log-transformed data); age groups, and underweight, normal weight and overweight/obesity statuses were compared using a one-way ANOVA test.

**Table 3:** Association between the mercury level (μg g^−1^) in hair and gender/occupational scenario.

Gender/Occupation	Not work at Medeber		Work at Medeber		*p*-Value ^1^
	AM ± SD ^2^	Median ± IQR ^3^	AM ± SD	Median ± IQR ^3^	
Females	1.0 ± 2.3	0.14 ± 0.41	3.6 ± 6.0	0.6 ± 4.1	0.010
Males	0.22 ± 0.28	0.13 ± 0.18	0.24 ± 0.43	0.08 ± 0.21	0.492
*p*-value ^1^	0.323		<0.001		

^1^ All variables were compared using an unpaired *t*-test (natural log-transformed data). ^2^ Arithmetic mean (AM); ± standard deviation (SD). ^3^ IQR = interquartile range.

**Table 4:** Univariate statistical analysis in the studied population according to age and body mass index (BMI).

Variable	Pearson’s Correlation Coefficient ^1^	*p*-Value ^1^
Age	−0.003	0.974
Body mass index (BMI)	0.095	0.313

^1^ Natural log-transformed data.
